# Metal Carbide as A Light‐Harvesting and Anticoking Catalysis Support for Dry Reforming of Methane

**DOI:** 10.1002/gch2.201900067

**Published:** 2019-10-03

**Authors:** Kazu Takeda, Akira Yamaguchi, Yohei Cho, Oruganti Anjaneyulu, Takeshi Fujita, Hideki Abe, Masahiro Miyauchi

**Affiliations:** ^1^ Department of Materials Science and Engineering Tokyo Institute of Technology 2‐12‐1, Ookayama, Meguro Tokyo 152‐8552 Japan; ^2^ National Institute for Materials Science 1‐1, Namiki, Tsukuba Ibaraki 305‐0044 Japan; ^3^ School of Environmental Science and Engineering Kochi University of Technology 185 Miyanokuchi, Tosayamada, Kami Kochi 782‐8502 Japan

**Keywords:** anticoking, catalysis, dry reforming of methane, plasmonics, tantalum carbide

## Abstract

Dry reforming of methane (DRM) is one of the most attractive chemical reactions, since it converts global‐warming gases into valuable syngas including hydrogen and carbon monoxide. Numerous previous studies used metal oxides catalysis supports, such as Al_2_O_3_, but their operating temperature was very high and severe coking occurred and deteriorated their catalytic activities. The present study reports that a metal carbide like tantalum carbide (TaC) acts as a multifunctional catalyst support for the DRM reaction, including light‐harvesting properties for saving energy operation as well as an anticoking property for long‐term stability. Nickel nanoparticles loaded on tantalum carbide (Ni/TaC) are prepared by impregnation and reductive hydrogen treatment. TaC particles act as a light‐harvesting support to promote the DRM reaction by photon irradiation through plasmonic photothermal energy conversion in TaC. Furthermore, Ni/TaC exhibits an excellent long‐term anticoking property, as compared to Ni loaded on conventional metal oxide supports such as Al_2_O_3_ or Ta_2_O_5_. According to the sole gas condition's experiment, and secondary ion mass spectroscopy, the oxy‐carbide layer near the interface between TaC and Ni plays an essential role in imparting the efficient anticoking property of Ni/TaC.

## Graphical abstract

Tantalum carbide functions as a light‐harvesting and anticoking catalysis support for dry reforming of methane.

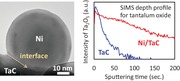

Methane (CH_4_) has recently become an attractive energy resource, since shale gas has been found to be stored as an abundant natural resource ideal for practical use. However, its combustion emits carbon dioxide (CO_2_), which causes global warming. Thus, the need for technologies that allow CO_2_ capturing and its utilization are urgent for sustainable energy and environmental management.^1^ Among various CO_2_ utilization methods, dry reforming of methane (DRM) is one of the most attractive chemical reactions, since it converts global‐warming gases into valuable syngas including hydrogen (H_2_) and carbon monoxide (CO)^2^ as follows

(1) $${\mathrm{CH}}_{4}+{\mathrm{CO}}_{2}=2H_{2}+2\mathrm{CO}\;\Delta H^{{}^{\circ}}{}_{298K}\;=247\;\mathrm{kJ/mol}$$

However, DRM is an endothermic reaction, thus requiring a high operating temperature, more than 1073 K, to achieve an efficient conversion or yield.^3^ Furthermore, intermediate carbon is deposited onto the catalyst during the DRM reaction, so‐called “coking,” which deteriorates catalytic activity.^4^ From a practical point of view, nickel (Ni)^5^ or cobalt (Co) catalysts are preferable promoters compared to noble metal catalysts,^6^ but their catalytic activities and anticoking properties for the DRM reaction are limited. Besides the development of metal catalysts, various catalysis supporting materials including metal oxides have been studied to solve the coking problem. For example, metal oxides consisting of alkaline‐earth elements such as magnesium oxide (MgO) were reported to suppress coking due to their strong CO_2_ adsorption property.^7^ Also, cerium oxide (CeO_2_) was studied as an anticoking catalysis support, owing to its efficient redox property at the interface between the metal catalyst and metal oxide.^8^

To lower the operating temperature of DRM, high‐quality photon energy can potentially assist the reaction using bandgap excitation of semiconductor photocatalysts,^9^ mid‐gap excitation of defective TiO_2_,^10^ and hot carrier generation in noble metal nanoparticles.^11^ In addition to these techniques, our group recently reported that tantalum carbide (TaC) promoted the DRM reaction involving a cobalt catalyst by using photon energy on the basis of photothermal conversion induced by surface‐localized plasmon resonance (SLPR) in TaC.^12^ Nagao and co‐workers investigated the plasmonic photothermal effect in various metal carbides and metal nitrides including TiC, ZrC, HfC, TaC, WC, TiN, ZrN, HfN, TaN, and WN by their theoretical approach.^13^ Then, the absorption efficiency (*Q*_abs_) of TaC was broadly ranged in visible light region, and its integral *Q*_abs_ value was higher than that of plasmonic silver (Ag) metal. This unique feature of TaC is owing to its strong and broad resonance, which makes it as a good sunlight harvester.

Herein, we have focused on a metal carbide that can be used as an anticoking catalysis support, as well as for its light‐harvesting property. Nickel nanoparticles loaded on tantalum carbide (Ni/TaC) were prepared and their catalytic properties were compared with those loaded on alumina and tantalum oxide (Ni/Al_2_O_3_ and Ni/Ta_2_O_5_) supports. In this study, we discuss anticoking properties of these catalysts with respect to their interface chemical structure between Ni particles and support materials.

Commercial TaC, γ‐Al_2_O_3_, and Ta_2_O_5_ powders were used as catalyst supports. Ni nanoparticles were loaded on these supports by impregnation in aqueous nickel nitrate solution and subsequent annealing treatment under hydrogen (H_2_) flow. Detailed experimental procedures are described in the Supporting Information. **Figure** **1** shows the characterization results of Ni/TaC particles. The X‐ray diffraction (XRD) pattern indicates the formation of crystalline metal Ni in addition to the strong diffraction peaks of TaC. According to the scanning electron microscope (SEM) image, pristine TaC particles exhibited smooth surface (Figure 1b), whereas small particles, less than 100 nm, appeared in Ni/TaC (Figure 1c). The transmission electron microscope (TEM) image revealed that the Ni nanoparticle with the size about 50 nm was attached onto TaC support (Figure 1d). The same structural analyses were conducted for Ni/Al_2_O_3_ and Ni/Ta_2_O_5_ and shown in Figures S1 and S2 in the Supporting Information, and Ni nanoparticles were successfully loaded onto these catalyst supports. X‐ray photoelectron spectroscopy (XPS) analysis also indicated that the chemical state of Ni nanoparticles was identical on various support materials (Figure S8b, Supporting Information).

**Figure 1:**
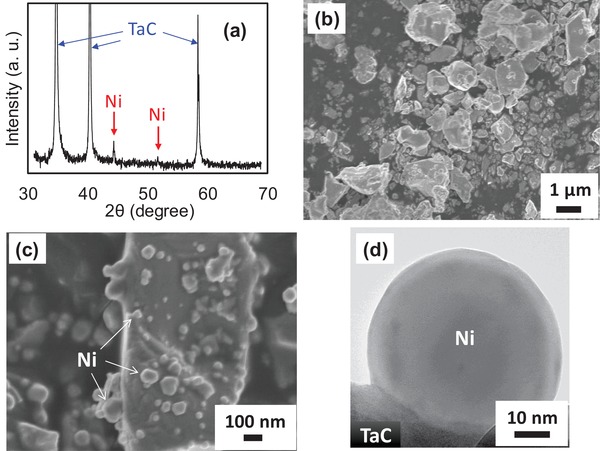
Powder XRD pattern for Ni/TaC (a), SEM images of pristine TaC (b) and Ni/TaC (c), and TEM image of Ni/TaC (d), respectively.

A catalytic test for the DRM reaction was performed, using a fixed bed reactor under flow of 1% of CH_4_ and CO_2_ mixture in argon (Ar) balance gas, where photons can be irradiated onto catalyst surface through a quartz window (Figure S3, Supporting Information). **Figure** **2** shows the H_2_ and CO yields at 773 K under light‐on or light‐off conditions. H_2_ production yield was relatively less than that of CO generation over all of the catalysts because of reverse water gas shift reaction. The H_2_ production yield of Ni/Al_2_O_3_ under dark condition was higher than the other catalysts, because its surface area (117.6 m^2^ g^−1^) was much higher than that of Ni/TaC (1.0 m^2^ g^−1^). It is noted that the light irradiation effect is the most significant in Ni/TaC among these catalysts. Panel (d) in Figure 2 shows UV–visible absorption spectra of these catalysts and spectrum of a xenon light source, which is close to the solar light spectrum. Ni/TaC has strong visible light absorption, which overlapped with the light irradiation. In this study, the photon‐driven DRM reaction is not simply explained by the bandgap excitation of catalysis supports, since both Al_2_O_3_ and Ta_2_O_5_ are ultra‐widegap semiconductors while TaC is a conductive material.^14^ Hot carriers generation in Ni nanoparticles is not dominant for this catalysis reaction, since Ni nanoparticles are identically loaded onto these support materials as shown in the Supporting Information (Figures S1 and S2, Supporting Information). According to the previous studies, some of the metal carbides can be excited through SLPR, and the visible light absorption in TaC is superior to those in early‐d‐metal carbides such as TiC, ZrC, HfC, or WC.^13^ Strong visible light absorption in Ni/TaC in this study is due to SLPR of TaC particles. The previous report indicated that the electric field near TaC involves a dipolar distribution and the temperature is considerably increased due to the SLPR excitation.^12^ These results indicate that TaC functioned as a light‐harvesting catalysis support for the DRM reaction. We have conducted the catalysis test under the specific diluted gas condition. However, our system uses photothermal effect, thus the catalytic behavior under high reactant pressures most likely depends on thermodynamical equilibrium, i.e., higher partial pressure causes a lower conversion and/or yield of DRM reaction.

**Figure 2:**
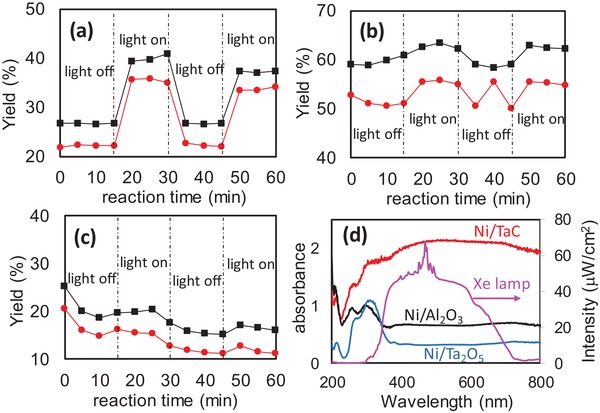
H_2_ (red circles) and CO production yield (black squares) at 773 K under xenon (Xe) light irradiation or dark conditions for Ni/TaC (a), Ni/Al_2_O_3_ (b), and Ni/Ta_2_O_5_ (c), respectively. Feed gas was CH_4_/CO_2_/Ar = 1/1/98 and flowed at 10 mL min^−1^. Panel (d) shows the absorption spectra of catalyst powder recorded by the diffuse‐reflectance method and the light spectrum of a 150 W xenon lamp (pink line).

Next, we evaluated the catalyst durability for the DRM reaction in the same reactor. **Figure** **3**a shows the time course of CH_4_ conversion using Ni catalysts on various catalyst supports for 24 h under dark condition. CH_4_ conversion on Ni/Al_2_O_3_ was drastically decreased after 500 min catalysis running, and the flow line of the reactor was plugged by coking. The DRM conversion activity in the presence of Ni/Ta_2_O_5_ was lower than those of the other catalysts. Interestingly, Ni/TaC exhibited stable DRM conversion activity. Similar trends were seen for CO_2_ conversion as well as H_2_ and CO production yields (Figure S4, Supporting Information). Figure 3b–d shows the SEM images of these catalysts after 24 h DRM reaction. It is noted that numerous carbon fibers appeared in either Ni/Al_2_O_3_ (Figure 3c) or Ni/Ta_2_O_5_ (Figure 3d), but fibrous carbon could not be seen in Ni/TaC (Figure 3b). Panel (e) in Figure 3 shows the amount of carbon of these catalysts after the DRM reaction analyzed by thermogravimetry and differential thermal analysis (TG‐DTA). Coking was significantly suppressed in the Ni/TaC catalyst. The turnover number after 24 h DRM reaction of Ni/TaC, which was calculated by the number of electrons required for H_2_ generation versus the loaded Ni catalyst amount, was 119. We also investigated the catalyst durability of Ni/TaC under Xe light irradiation at 773 K for 50 h, and DRM conversion and yield were sustained for a long term (Figure S5, Supporting Information). Conversions and/or yields under light irradiation were similar to those under dark condition. In our system, the light absorber is metallic carbide unlike semiconductor photocatalysts, thus photon energy can be converted into thermal energy. Therefore, it is reasonable that the trend under dark condition was similar to that under light irradiation. The previous study suggested that the C–H dissociation in CH_4_ is the rate‐determining step as was widely accepted for DRM on Ni thermal catalysts.^15^ It is also known that coking proceeds after the cracking of the CH_4_ molecule,^16^ thus, the oxidation process of residual carbon is very important for an efficient anticoking function.

**Figure 3:**
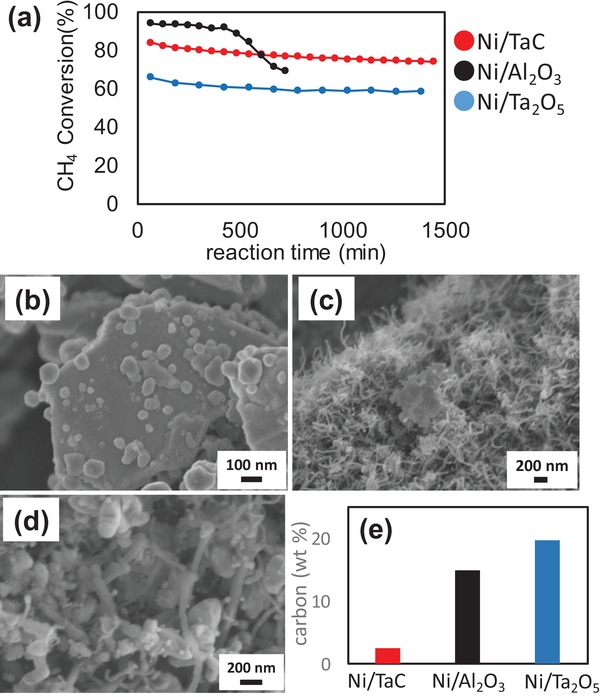
Durability test under DRM condition at 973 K. a) Change in CH_4_ conversion of Ni catalysts on various support materials. SEM images after the DRM reaction of b) Ni/TaC, c) Ni/Al_2_O_3_, d) Ni/Ta_2_O_5_. Panel e) demonstrates carbon amount of these samples after the DRM reaction analyzed by TG‐DTA.

Here, we discuss the mass transport process of oxygen species in the Ni/TaC catalyst under the DRM reaction. First, we evaluated the catalytic activity under sole CH_4_ or CO_2_ conditions and analyzed the change of the oxygen amount in the catalysts by energy dispersive X‐ray spectrometry (EDS), as shown in **Figure** **4**. In this experiment, 1% CH_4_ in Ar gas was flowed and the temperature was increased from room temperature (295 K) to 973 K. After that, EDS analysis was performed, then the catalyst was re‐introduced into a reactor for the test under CO_2_ gas without CH_4_ condition. Regarding the Ni/TaC catalyst under sole CH_4_ condition (Figure 4a), H_2_ generation was about twice of the CO concentration, indicating these products originated from CH_4_. Interestingly, CO was produced even under sole CH_4_ condition, where no oxygen species were introduced in the gas phase. The chemical composition of the oxygen/tantalum (O/Ta) ratio was also investigated by EDS before and after CH_4_ flow condition plotted as red circles in Figure 4a. The O/Ta ratio before the test was 0.8, which decreased to 0.3 after sole CH_4_ treatment. These results strongly imply that oxygen in the produced CO molecules originated from some oxygen species in the Ni/TaC catalyst. When the catalyst was exposed under sole CO_2_ condition, the O/Ta ratio was recovered to 0.45 and the produced CO was equivalent to CO_2_ conversion. These oxygen amount changes in the Ni/TaC catalyst and the CO production trend were seen in neither Ni/Al_2_O_3_ (Figure 4b) nor Ni/Ta_2_O_5_ (Figure S6, Supporting Information). These results strongly imply that oxygen species in Ni/TaC play an important role for its anticoking property. Previous studies also suggested that the oxygen species in molybdenum carbide participated in the redox reaction of DRM.^17^ In this study, we expected that some oxygen species would be formed at the interface between Ni and TaC, since the catalyst was prepared by an H_2_ reduction treatment of amorphous nickel oxide nanoparticles, where oxygen species in these nanoparticles would partially oxidize TaC near their interface, as speculated in Figure S7a in the Supporting Information.

**Figure 4:**
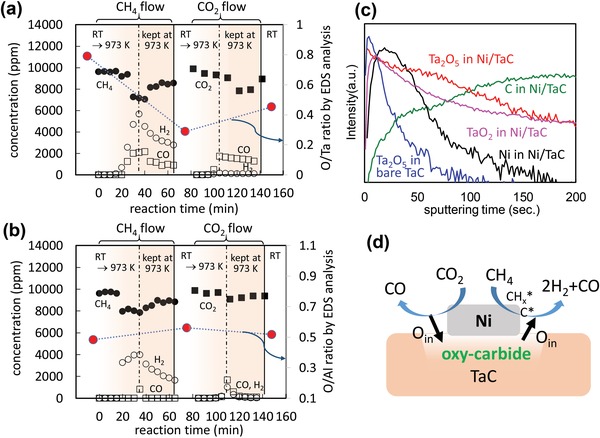
Sole gas reactivity test for Ni/TaC a) and Ni/Al_2_O_3_ b), where chemical composition was analyzed by EDS (red circles). Panel c) shows the SIMS depth profile of Ni film coated TaC pellet under the same preparation procedure with powder catalyst. d) Expected catalysis mechanism at the interface.

We investigated the interface structure between Ni and TaC by secondary ion mass spectrometry (SIMS) using the thin film model of the catalyst structure. The thin film of NiO was coated on a bulk TaC pellet by a DC magnetron sputtering with the thickness of 50 nm, which was close to the particles size of Ni as shown in Figures 1d or 3b. The NiO film on the TaC substrate was annealed under the same H_2_ flow condition with particle preparation. XRD patterns of this film before and after H_2_ treatment, shown in Figure S7b in the Supporting Information, indicate that the metallic Ni film was successfully coated on a TaC pellet. Figure 4c shows the depth profile of this film for each mass fragment by SIMS analysis. The blue line shows the profile of the Ta_2_O_5_ fragment in a bare TaC pellet as a control group. By comparing the fragment profile of Ta_2_O_5_ in the Ni/TaC film (red line) with that in the pristine TaC film (blue line), the oxygen species were found to diffuse deeper from the surface in the Ni/TaC film sample. The black line shows the profile of the metal Ni fragment, and it ended at the sputtering time of 180 s, corresponding to its thickness of 50 nm. These results revealed that oxygen species were diffused into the TaC side. These oxygen species would be formed during the annealing treatment under H_2_ to reduce nickel oxide. These oxy‐carbide species near the interface would play an important role in the efficient and stable DRM reaction reported in this study.

Although oxygen atoms in metal oxides would be more abundant than those in TaC, Al_2_O_3_ and Ta_2_O_5_ supports did not contribute to their anticoking property. We suppose that the chemical state of oxygen species at the interfacial oxy‐carbide in the Ni/TaC catalyst is different from those in densely packed metal oxides. Therefore, we conducted the X‐ray photoelectron spectroscopy analysis on Ni/TaC and Ni/Ta_2_O_5_ samples to discuss their oxygen ions states (Figure S8, Supporting Information). The O‐1s peak of Ni/TaC was shifted toward higher binding energy as compared to that of Ta_2_O_5_. Previous studies also reported the similar chemical shift of the O‐1s peak in defective metal oxides.^18^ These oxygen defects are known to be active sites for the reduction of CO_2_ or nitro‐compounds.^19^ According to our XPS analysis on the Ta‐4f core level (Figure S8c, Supporting Information), the Ni/TaC catalyst contains Ta^4+^ species and Ta—C bonding after sole CH_4_ gas exposure condition. Also, the mass signal for the TaO_2_ fragment was detected in Ni/TaC by SIMS analysis in addition to that of the Ta_2_O_5_ fragment. These Ta^4+^ species would be formed by the release of oxygen ions from the oxy‐carbide layer to CH_4_, then residual carbon species were formed near the interface of the catalyst. Figure 4d shows the schematic diagram to depict the reaction scheme of the present Ni/TaC catalyst. Oxygen species at the interfacial oxy‐carbide layer (O_in_) act as a redox mediator for CO_2_ reduction into CO (CO_2_ → CO + O_in_) and CH_4_ oxidation (CH_4_ + O_in_ → 2H_2_ + CO). CH_4_ oxidation and CO_2_ reduction would be balanced on our Ni/TaC; thus, the long‐term efficiency of the DRM reaction was sustained, as shown in Figure 3 and Figure S5 in the Supporting Information. A similar redox cycle for the DRM reaction was reported in the cerium and/or lanthanum oxide support.^20^

In conclusion, the TaC catalysis support is multifunctional due to its light‐harvesting property for saving energy operation as well as an anticoking property for long‐term stability of CH_4_ conversion reaction. The light‐harvesting property is driven by the SLPR in TaC under visible light irradiation. The anticoking property is imparted to Ni/TaC by the interfacial oxygen species between the Ni catalyst and TaC support. The interfacial oxy‐carbide layer in TaC act as a redox mediator for CO_2_ reduction and CH_4_ oxidation, thus the residual carbon atoms were efficiently oxidized into CO in the Ni/TaC catalyst. The findings of this study demonstrate that metal carbide can be used as an efficient catalyst support for not only dry reforming but also for partial oxidation or steam reforming of CH_4_ reactions.

## Conflict of Interest

The authors declare no conflict of interest.

## Supporting information

Supplementary

Supplementary
