# Supplementary material for: Metal Carbide as A Light‐Harvesting and Anticoking Catalysis Support for Dry Reforming of Methane
Source: Glob Chall. 2019 Oct 3;4(1):1900067. doi: 10.1002/gch2.201900067 (PMC6957020; doi:10.1002/gch2.201900067)
Supplement: Supplementary file 1 — Supplementary [file GCH2-4-1900067-s001.pdf]

Copyright WILEY-VCH Verlag GmbH & Co. KGaA, 69469 Weinheim, Germany, 2019.

Supporting Information

for *Global Challenges,* DOI: 10.1002/gch2.201900067

Metal Carbide as A Light-Harvesting and Anticoking Catalysis Support for Dry Reforming of Methane

*Kazu Takeda, Akira Yamaguchi, Yohei Cho, Oruganti Anjaneyulu, Takeshi Fujita, Hideki Abe, and Masahiro Miyauchi**

Copyright WILEY-VCH Verlag GmbH & Co. KGaA, 69469 Weinheim, Germany, 2019.

Supporting Information

Metal Carbide as A Light-harvesting and Anti-coking Catalysis Support for Dry Reforming of Methane

Kazu Takeda, Akira Yamaguchi, Yohei Cho, Oruganti Anjaneyulu, Takeshi Fujita, Hideki Abe, and Masahiro Miyauchi *

Contents:

Experimental method

Figure S1 Characterization of Ni/Al_2O_3.

Figure S2 Characterization of Ni/Ta_2O_5.

Figure S3 Experimental setup for catalysis evaluation.

Figure S4 Durability test under DRM condition.

Figure S5 Long term stability of DRM reaction over Ni/Ta_2O_5. under light irradiation.

Figure S6 Sole gas reactivity test for Ni/Ta_2O_5.

Figure S7 Schematic illustration of the interfacial structure between Ni and TaC (a) and XRD patterns of thin film model (b).

Figure S8 XPS spectra.

Experimental method

Nickel nitrate hexahydrate, tantalum carbide, and tantalum pentoxide were purchased from Wako Pure Chemical Corporation. Aluminum oxide was purchased from Sigma-Aldrich. The tantalum carbide tablet and Ni target were purchased from JAPAN PURE CHEMICAL CO. LTD. They were used without further purification.

Powder catalyst samples were prepared by impregnation method. 0.0989 g of nickel nitrate hexahydrate was dissolved in 10 mL deionized water, in which 0.180 g support powder (tantalum carbide, tantalum pentoxide or aluminum oxide) was added. After 24 h impregnation, they were dried at 383 K for one night. The obtained powder was put into an aluminum boat and calcined at 623 K for 4 h under Ar atmosphere flow. Next, grained powder was calcined again at 973 K for 4 h under 1 % H_2 in Ar gas flow (10mL/ min).

The thin film model was prepared by DC magnetron sputtering. A TaC pellet was used as a substrate, whose surface was mechanically polished. A nickel oxide film was deposited by DC magnetron sputtering (Vacuum Device, MSP-30T) under oxidative atmosphere. The deposition condition was 1 Pa (Ar:O_2= 2:1) with 300 mA current for 2 min. Then, the film was calcined under the same condition with the procedure of powder catalyst preparation (at 623 K 4 h under Ar and at 973 K for 4 h under 1 % hydrogen flow).

Characterization was conducted using X-ray diffraction (Rigaku, Smartlab), SEM with an EDS analyzer (Keyence, VE-9800) or FE-SEM (JEOL Ltd., JFM-7500F), TEM (JEOL Ltd., JEM-2010), UV-vis with a diffuse reflection unit (JASCO Corporation, V-770), TG-DTA (NETZSCH, STA2500 Regulus), SIMS (ION-TOF, TOF-SIMS 5-100-AD), and XPS (ULVAC-PHI, Versa Probe). The scan rate of XRD was 4 degree/ min. The acceleration voltage was 3 kV (SEM) and 200 kV (TEM), respectively. TG-DTA analysis was conducted under 20 mL/ min under air flow and the temperature was increased to 1073 K with the increasing rate at 20 K/ min. The depth profile by SIMS was recorded with Bi^{++}_3 as primary ions and the Cs ion as the sputtering source with 60 keV beam voltage. The acceleration voltage of EDS was 8 kV. The X-ray source of XPS was Mg kα and pass energy was 23.5 eV. Obtained XPS spectra were calibrated with the Au-4$f_{7/2}$ peak (83.5 eV).

The catalytic performance test was performed in a flow reactor depicted in Figure S3. Each catalyst was 5 mg and set on an alumina ceramic cup in the reactor. The gas flow rate was 10 mL/ min and gas composition was CH_4:CO_2:Ar = 1:1:98. The temperature increasing rate was 20 K/min. The gas concentration during the test was analyzed by micro gas chromatography (Inficon, 3000 MicroGC). For the light irradiation test, a 150 W xenon lamp was used as a light source. The distance between the window and edge of fiber of light source was 1.6 cm. The temperature was 773 K for 1 h. Light on or off condition was changed every 15 min. In the durability test, temperature was 973 K for 24 h. In these tests, gas conversion or yield were calculated by equations (A) and (B).

The sole gas reactivity test was conducted in the same reactor, as described above. The gas flow rate was 10 mL/ min and gas composition was CH_4: Ar=1: 99 or CO_2: Ar=1: 99. The temperature increasing rate was 20 K/ min. In this experiment, 1 % CH_4 gas was flowed and temperature was increased from room temperature (295 K) to 973 K. After that, EDS analysis was performed, and then the catalyst was re-introduced into a reactor for the test under CO_2 gas without CH_4 condition.

$$\text{Conversion (\%)} = 100 \times \frac{\text{Inlet gas amount (ppm)} - \text{Outlet gas amount (ppm)}}{\text{Inlet gas amount (ppm)}} \quad \text{(A)}$$

$$\text{Yield (\%)} = 100 \times \frac{\text{Product gas amount (ppm)}}{\text{Ideal product gas amount (ppm)}}$$

$$= 100 \times \frac{\text{Product gas amount (ppm)}}{\text{20000 (ppm)}} \quad \text{(B)}$$

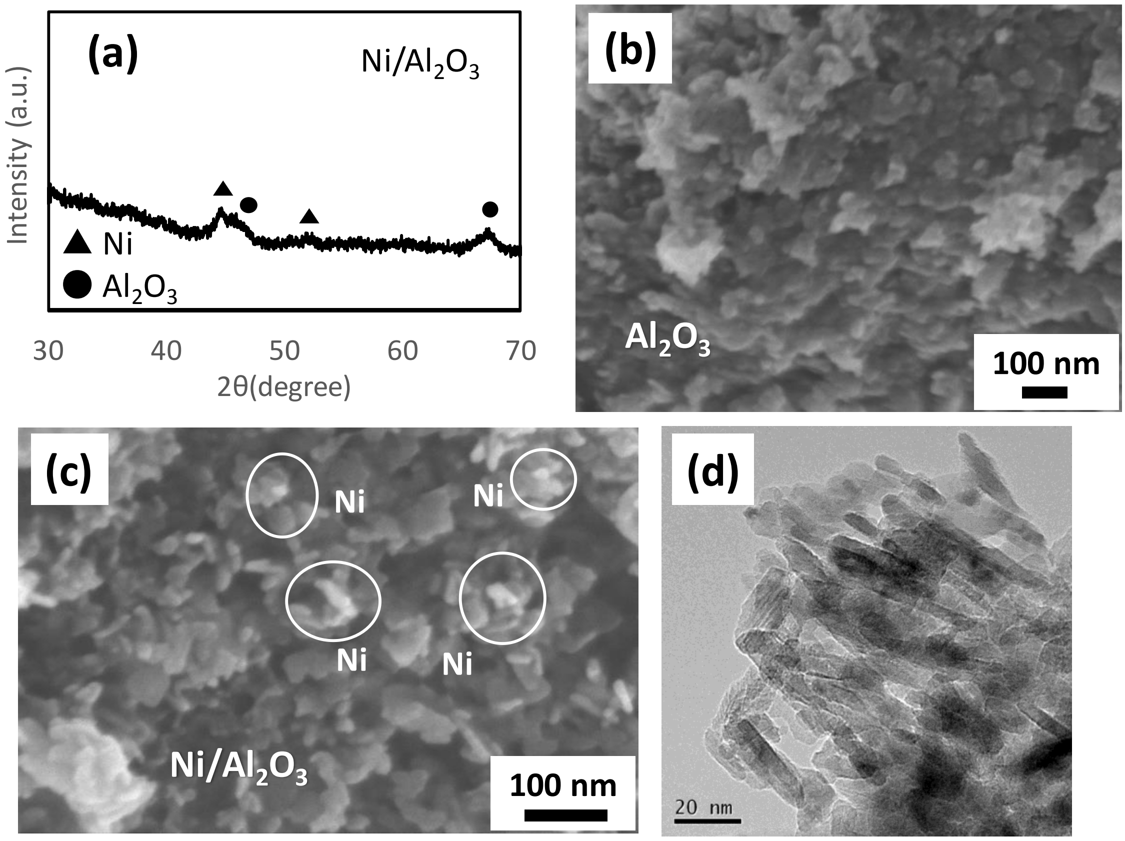

Figure S1. Characterization of Ni/Al_2O_3. (a) XRD pattern, (b) SEM image of pristine Al_2O_3 (c) SEM image of Ni/Al_2O_3, and (d)TEM image of Ni/Al_2O_3, respectively.

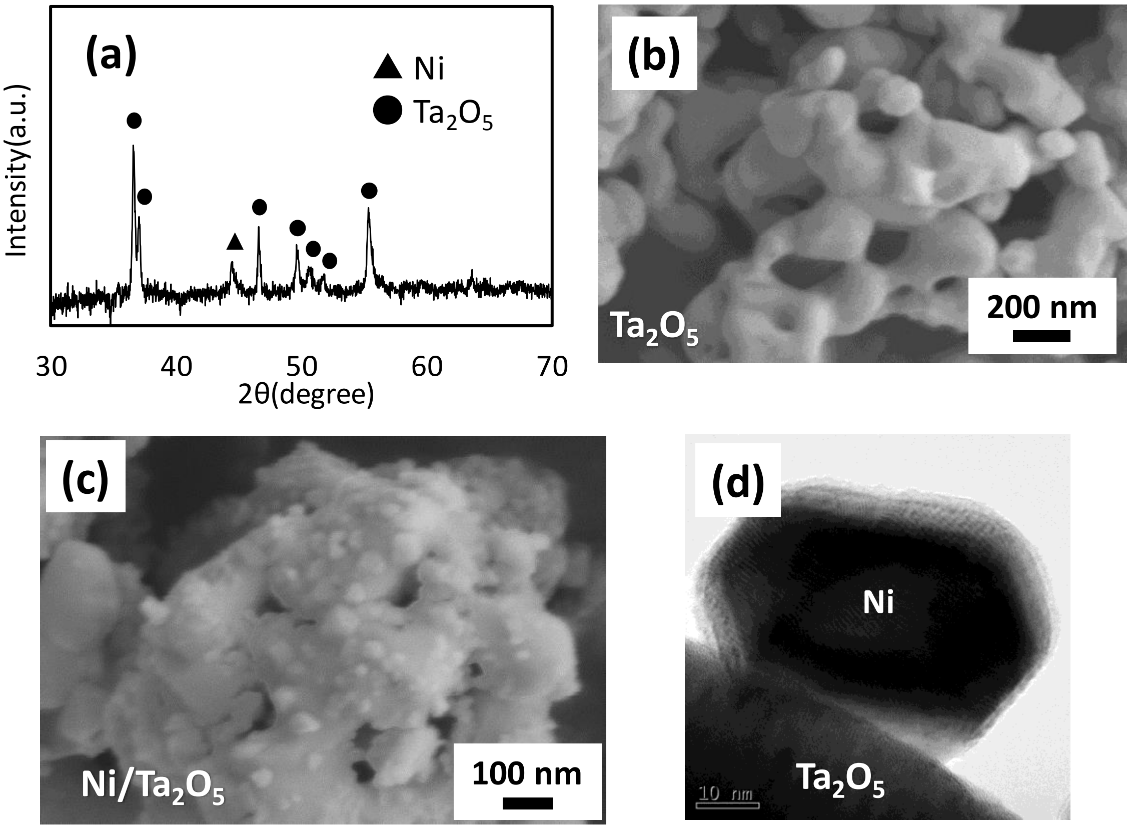

Figure S2. Characterization of Ni/Ta_2O_5. (a) XRD pattern, (b) SEM image of pristine Ta_2O_5 (c), SEM image of Ni/Ta_2O_5, and (d) TEM image of Ni/Ta_2O_5.

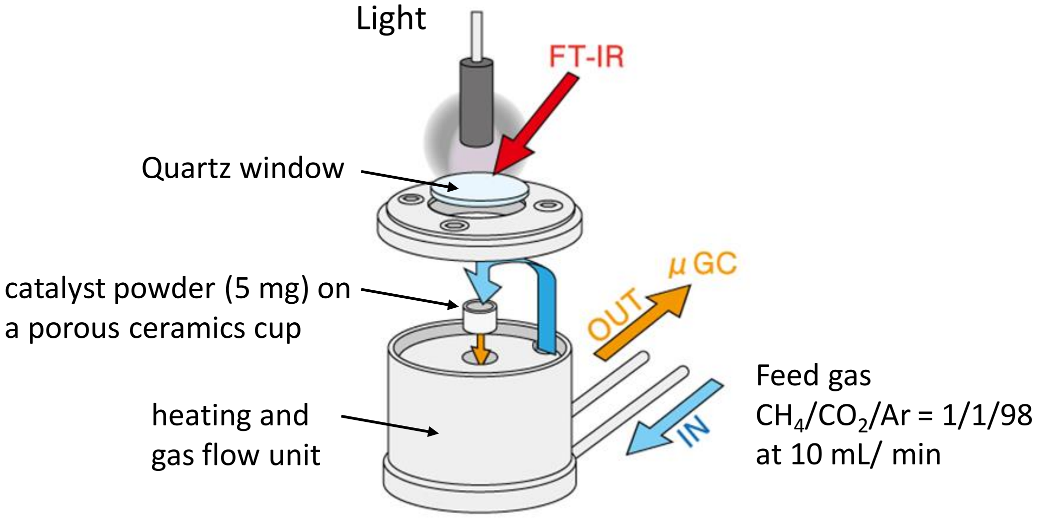

Figure S3. Flow reactor evaluation system for catalytic performance.

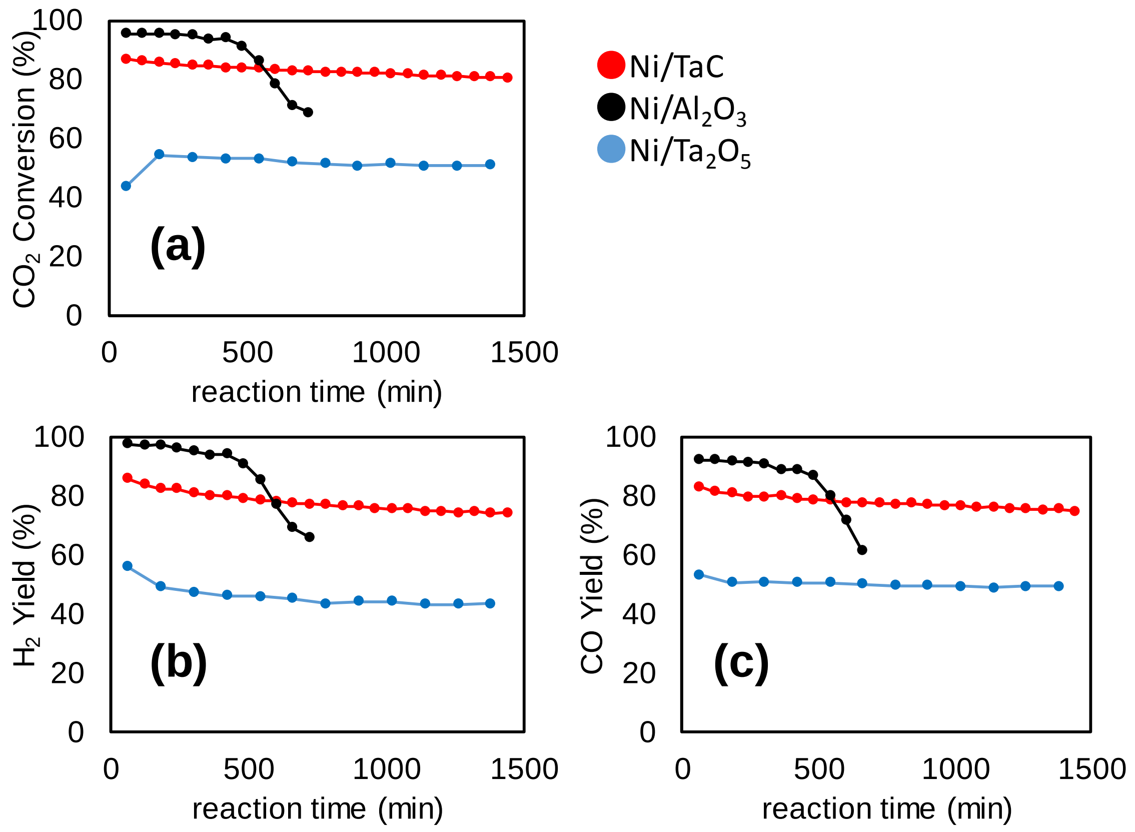

Figure S4. Durability test under DRM condition at 973 K for Ni catalysts on various support materials. (a) CO_2 conversion, (b) H_2 yield, and (c) CO yield, respectively.

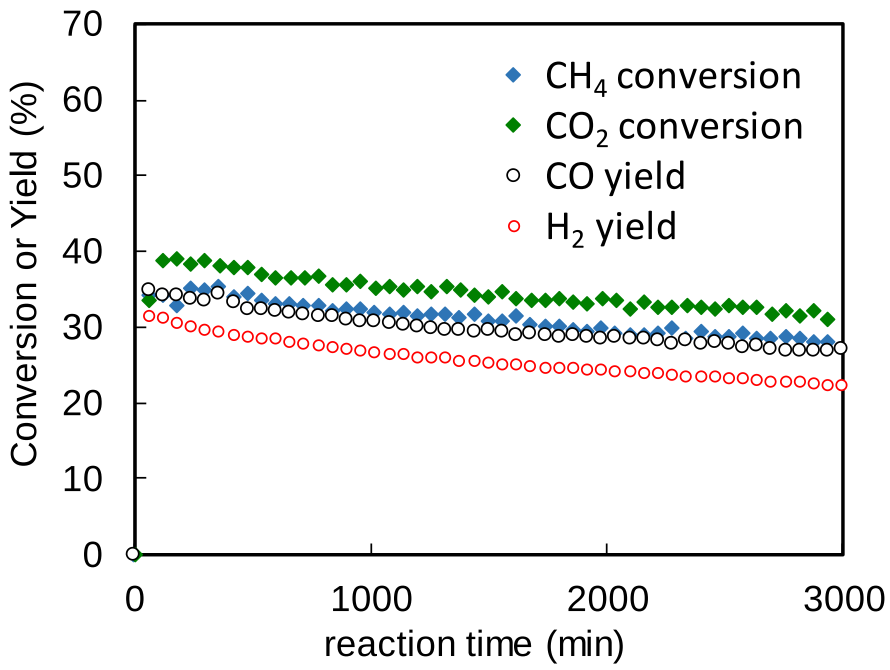

Figure S5. Long term durability of Ni/TaC for DRM catalysis reaction under Xe lamp irradiation at 773 K for 50 h.

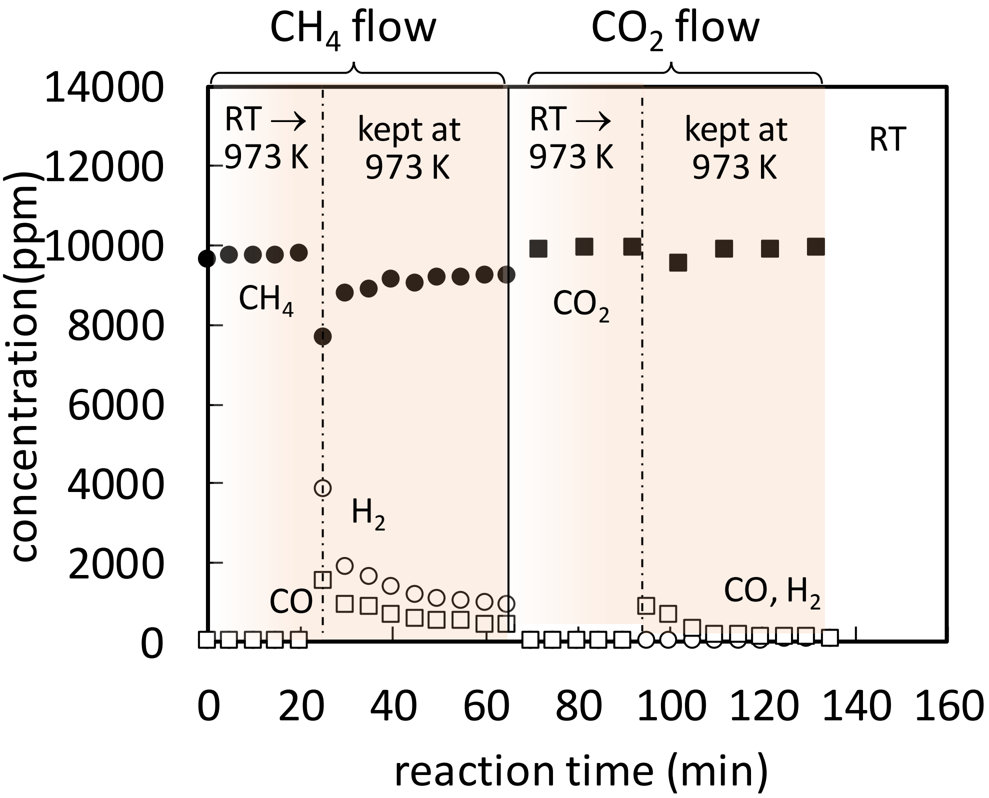

Figure S6. Sole gas reactivity test for Ni/Ta_2O_5.

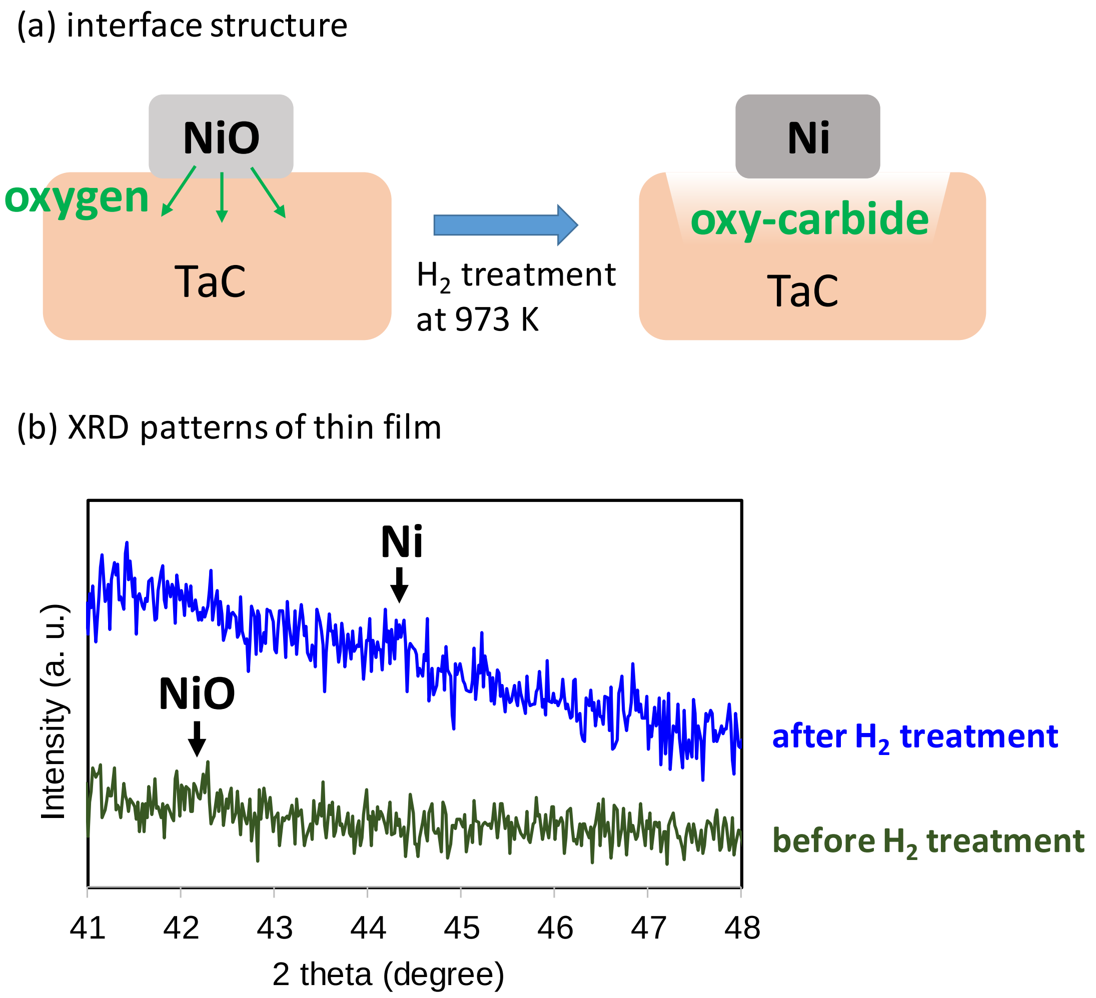

Figure S7. Schematic illustration of the interfacial structure between Ni and TaC (a). XRD patterns for thin film model before and after H_2 treatment (b).

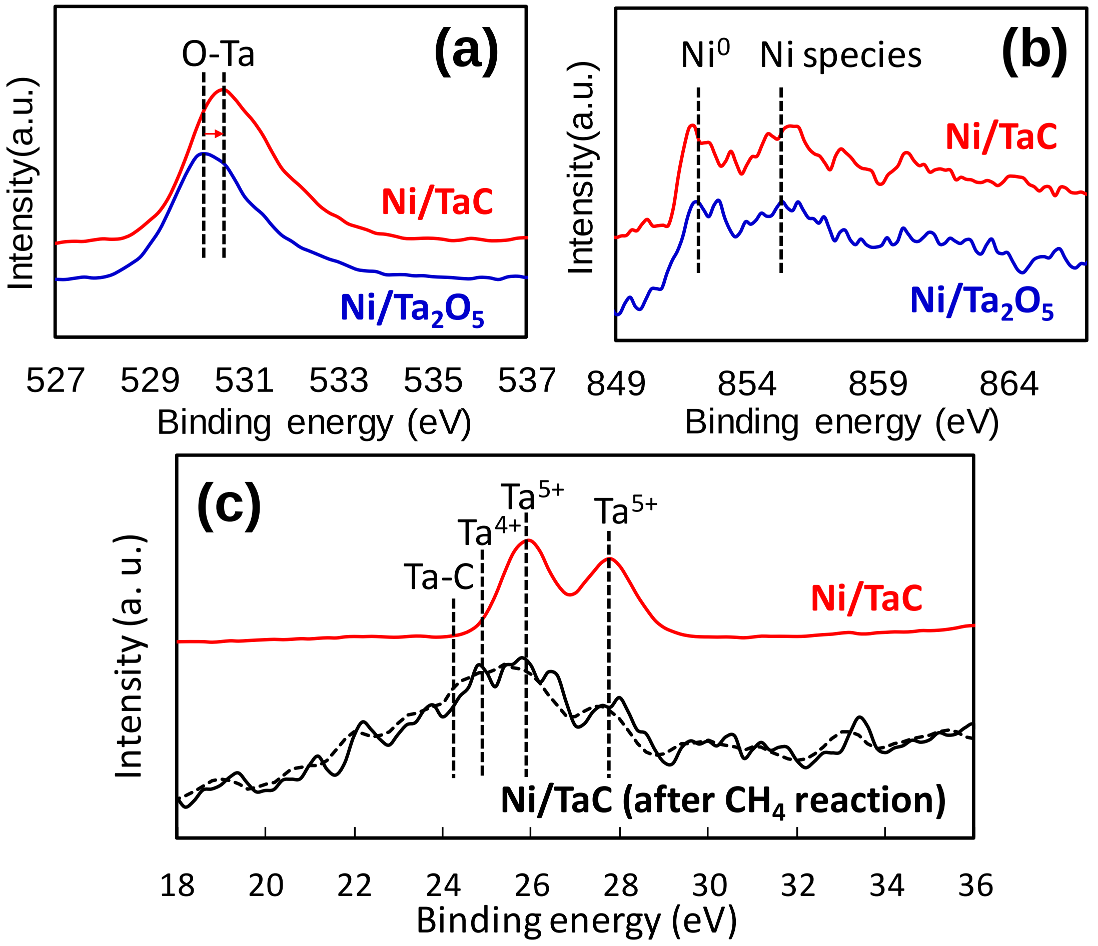

Figure S8. XPS spectra of Ni/TaC and Ni/Ta_2O_5 for O-1s (a) and Ni-2p (b) core levels, respectively. Panel (c) shows Ta-4f spectra of Ni/TaC and that reacted with CH_4 for 24 h at 1073 K.
